# Having and eating the cake? The centralisation paradox in decentralised healthcare governance: a scoping review

**DOI:** 10.1186/s12913-026-15162-1

**Published:** 2026-07-24

**Authors:** Mikael Ohrling

**Affiliations:** 1 Department of Learning, Informatics, Management and Ethics, Medical Management Centre, Karolinska Institutet, Stockholm, 171 77 Sweden; 2 Stockholm Health Care Services, Region Stockholm, Sweden

**Keywords:** Decentralisation, Healthcare governance, Decision space, Centralisation paradox, Organisational capacity

## Abstract

**Background:**

Decentralisation in healthcare governance is widely promoted to enhance managerial autonomy, responsiveness, and performance. However, empirical evidence suggests that managers operating in decentralised systems continue to rely on centrally coordinated organisational structures. This interdependence remains insufficiently conceptualised in existing research.

**Methods:**

A scoping review was conducted to synthesise empirical studies examining how central coordination in organisational systems shapes managers’ effective decision space in decentralised healthcare organisations. Searches were performed in PubMed and CINAHL, complemented by snowballing. The review followed PRISMA-ScR guidelines. Data were analysed using a conceptual synthesis approach informed by paradox theory and the decision space framework.

**Findings:**

Four interrelated themes were identified: (1) delegated authority and experienced autonomy, (2) the role of organisational capacity in enabling decision space, (3) persistent central coordination within decentralised systems, and (4) autonomy dependent on coordination. The findings show that while decentralisation expands formal decision-making authority, the effective exercise of this authority depends on centrally coordinated enabling systems, including digital infrastructure, administrative support, and financial management. This interdependence reflects a consistent pattern across contexts.

**Conclusions:**

The study proposes the concept of a *centralisation paradox*, demonstrating that managerial autonomy in decentralised healthcare governance is contingent upon central coordination. By extending the decision space model, the study proposes a dual-domain conceptualisation distinguishing between professional decision-making and enabling systems. This reframing advances theoretical understanding of decentralised governance and offers practical insights for designing healthcare organisations that balance local autonomy with system-wide coordination.

**Supplementary Information:**

The online version contains supplementary material available at 10.1186/s12913-026-15162-1.

## Introduction

Decentralisation in healthcare governance is intended to increase managerial autonomy; however, managers often report a simultaneous need for stronger central coordination to support local service delivery [1–3]. Autonomy, defined as the perceived and actual discretion to make decisions and influence work processes, is a fundamental condition for effective managerial practice in healthcare [4]. In increasingly complex and resource-constrained health systems, managers are expected to ensure quality, patient safety, financial sustainability, and workforce well-being, often under intensified regulation and central control [5]. The extent of their available “decision space,” however, varies across contexts and governance arrangements. Evidence suggests that autonomy is not only a structural feature of organisational design but also a critical resource associated with managerial well-being, motivation, and performance [6–9].

Decentralisation has long been promoted as a strategy to enhance autonomy and thereby improve responsiveness, efficiency, and organisational performance. By delegating decision-making authority to managers closer to service delivery, decentralised structures are expected to facilitate local adaptation to patient needs [1–3, 10]. In many organisations, this has been implemented through clinical directorates and hybrid management models, in which clinicians assume managerial responsibilities while maintaining professional legitimacy [11–13]. These roles are intended to integrate professional and managerial logics and support improvements in care quality and service delivery [6, 14].

Empirical studies indicate that managers generally value the autonomy associated with delegated authority [13–16]. Such local decision-making enables adaptation to contextual needs, flexible resource allocation, and timely responses to changing clinical demands [13]. Research on hospital governance and hybrid roles shows that clinicians in managerial positions perceive decentralised decision space as supporting context-sensitive management and improved coordination of care [17, 18]. Studies from European and Nordic systems further suggest that delegated authority promotes organisational responsiveness, professional engagement, and service innovation, although its effective use depends on supportive structures and resources [3, 11, 13, 14, 17]. During the COVID-19 pandemic, decentralised arrangements facilitated rapid organisational responses when managers had sufficient decision space and capacity [19].

Despite these benefits, decentralised organisations remain dependent on central coordination [16, 20–22]. Managers frequently rely on centrally organised infrastructures, such as digital systems, financial frameworks, and administrative support to enact local decisions [14]. These systems provide the organisational capacity required for coordination across units and for implementing managerial decisions. Managers therefore value both delegated authority and the conditions that enable its effective use [3, 13, 17, 18].

Recent research suggests that decentralisation does not reduce the need for central coordination but may instead increase it [14, 18, 23]. In practice, managers often advocate greater local autonomy while simultaneously requesting stronger coordination in specific organisational domains [14, 21]. This indicates that the relationship between centralisation and decentralisation is more complex than traditionally assumed.

While decentralisation aims to expand managerial autonomy, its effective exercise depends on centrally coordinated organisational systems. Managers seek discretion in professional decision-making while relying on central support structures [14]. Although prior research has identified tensions between central control and local autonomy, these have rarely been conceptualised explicitly as a paradox [23, 24]. Drawing on paradox theory, this paper introduces the concept of a *centralisation paradox* to capture the interdependence between decentralised managerial autonomy and centrally coordinated organisational systems [25].

Despite extensive research on decentralisation and hybrid management, limited attention has been given to how this paradox is perceived by clinical directors within the decision space framework [14]. Existing applications of decentralisation frameworks primarily focus on delegated authority and institutional conditions shaping capacity and accountability at the system level [1, 2, 26]. Adaptations for service delivery emphasise formal delegated authority at the clinical level, supported by stratified systems theory [10, 27], but tend to treat decentralisation as a structural characteristic. Less attention has been paid to how centrally coordinated systems, such as administrative structures, digital infrastructure, and financial management, may enable rather than constrain the exercise of delegated authority [28].

This suggests a mechanism within decentralised governance in which managerial autonomy is contingent upon centrally coordinated organisational systems. Understanding this relationship is essential for refining theoretical models of decentralisation and explaining how managers enact decision space in practice.

## Aim

Against this background, the aim of this paper is to contribute to theory development in research on decentralised healthcare governance in service delivery by conceptually extending the modified decision space model through the integration of the concept of the *centralisation paradox*. By introducing this concept in relation to findings from previous empirical studies of clinical directors’ experiences of decision space and autonomy, the paper seeks to advance theoretical understanding of how decentralised governance operates in practice and how managerial autonomy relates to organisational coordination within complex healthcare organisations.

### Theoretical background

#### Paradox theory and decentralised healthcare governance

Paradox theory has emerged as a prominent perspective for understanding persistent tensions within organisations. Rather than conceptualising organisational challenges as problems that can be resolved through trade-offs, paradox theory views many organisational conditions as characterised by contradictory yet interdependent elements that coexist over time [25, 28]. Such tensions arise when organisations are required to pursue competing demands simultaneously, making them endure rather than resolvable [25, 29].

Paradoxes are typically defined as situations in which elements appear logically inconsistent but remain mutually related and persist over time [30]. Common examples include flexibility versus control, exploration versus exploitation, and collaboration versus competition. In organisational settings, these tensions often reflect complex institutional environments, competing stakeholder expectations, and the need to balance short-term and long-term objectives [25, 29, 31].

In healthcare systems, paradoxical tensions are particularly pronounced due to the coexistence of professional autonomy and organisational governance structures [18, 24]. Professional bureaucracies must simultaneously enable expert-driven decision-making at the local level while ensuring coordination, standardisation, and accountability across organisational units [24]. As a result, decentralisation and central coordination are not opposing governance models but interdependent mechanisms that must be enacted simultaneously [28, 32, 33].

This perspective is especially relevant for understanding the core tension addressed in this paper: the coexistence of decentralised managerial autonomy and centrally coordinated organisational systems. While decentralisation is intended to expand local decision-making authority, its practical enactment often depends on infrastructures and support functions organised at higher levels of the system. A paradox lens thus enables the analysis of decentralised governance not as a linear shift of authority, but as a dynamic and ongoing interplay between autonomy and coordination.

#### Decentralisation and the decision space framework

Decentralisation in healthcare governance refers to the delegation of authority and responsibility from higher levels of governance to managers closer to service delivery. The underlying rationale is that proximity to clinical practice enables more context-sensitive decision-making and improved organisational performance [7].

Bossert’s decision space model conceptualises decentralisation as the range of effective choice available to local managers within a governance structure [1]. The model highlights that decentralisation varies across functional domains and is shaped by the degree of discretion granted by central authorities. Subsequent developments have extended this framework by emphasising that the effects of decentralisation depend not only on formal delegation but also on managerial capacity and accountability mechanisms [10].

**Fig. 1:**
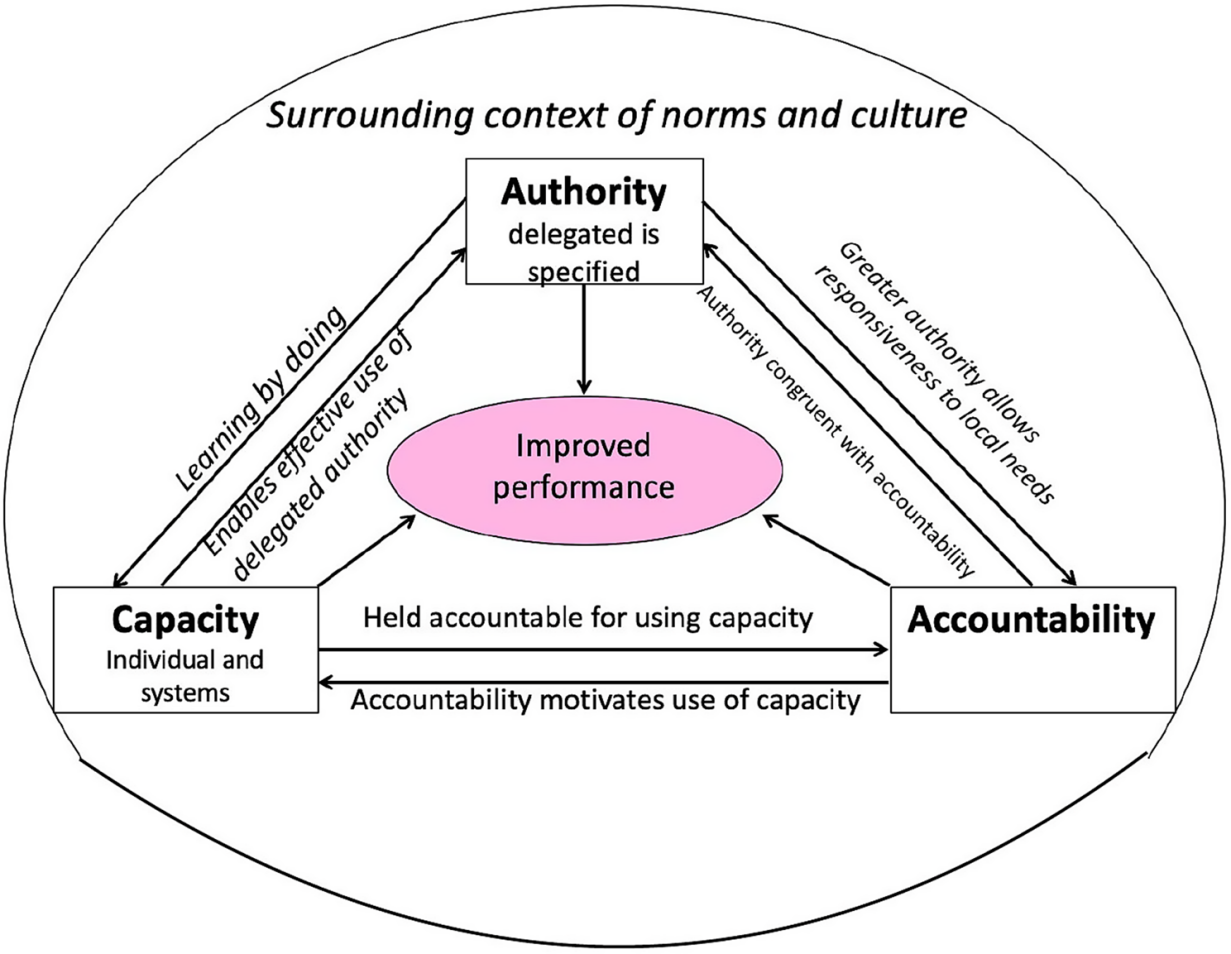
Modified decision space model illustrating effective decentralisation through authority delegation, capacity development and accountability in healthcare service delivery

In its modified form for healthcare service delivery organisations (Fig. 1), the model conceptualises decentralisation as a dynamic relationship between three interdependent elements: delegated authority, managerial capacity (at both individual and organisational levels), and accountability [10]. Delegated authority defines the formal scope of decision-making, capacity determines the ability to exercise this authority effectively, and accountability structures regulate and guide managerial action. These elements are embedded within a broader organisational context shaped by norms, culture, and systems, which may enable or constrain managerial practice [28, 32].

While the decision space framework provides a robust model for analysing the structural conditions of decentralisation, it has primarily been applied to examine the distribution of authority and the institutional factors influencing its use. Less attention has been paid to how organisational systems, often coordinated centrally, condition the practical enactment of delegated authority. In particular, the model does not explicitly conceptualise the interdependence between local autonomy and the centrally organised infrastructures that make such autonomy actionable for the clinical directors in practice.

#### Hybrid management and the enactment of decentralisation

The integration of clinicians into managerial roles represents a key mechanism for operationalising decentralisation in healthcare organisations [13, 34]. Hybrid managers combine professional expertise with managerial responsibilities and are expected to bridge professional and organisational logics [12]. Clinical directorates exemplify this model by strengthening managerial capacity at the service delivery level while preserving professional legitimacy in clinical decision-making [35, 36].

However, hybrid managerial roles are inherently situated at the intersection of multiple organisational demands. Clinicians in managerial positions are expected to exercise professional autonomy while simultaneously ensuring compliance with organisational goals, regulatory requirements, and system-wide coordination. When such roles exist across multiple organisational levels, ambiguities in authority and responsibility may further complicate coordination and decision-making processes [14].

From a paradox perspective, hybrid management can be understood as a site where tensions between autonomy and coordination become particularly salient. Hybrid managers must navigate and enact decentralised authority within organisational systems that are often centrally structured. This positions them at the core of the dynamic interplay between local discretion and system-level coordination.

#### Towards a centralisation paradox in decentralised healthcare governance

Taken together, paradox theory and the decision space framework offer complementary perspectives for analysing decentralised healthcare governance. The decision space model explains how authority is formally distributed and exercised, while paradox theory provides a lens for understanding the persistent tensions that arise in this process.

Integrating these perspectives makes it possible to conceptualise decentralisation not only as a structural arrangement but as a dynamic and interdependent relationship between managerial autonomy and organisational coordination. In this context, the effective exercise of decision space is contingent upon organisational systems that are often centrally coordinated, including administrative structures, digital infrastructures, and financial management systems.

## Methods

### Study design

A scoping review methodology was employed to identify, summarise, and synthesise empirical studies that contribute to understanding how central coordination in enabling organisational systems shapes managers’ effective decision space in decentralised healthcare organisations. Scoping review methodology was considered appropriate given the exploratory and theory-developing aim of the study, allowing for the inclusion of diverse study designs and a broad conceptual scope. The review methodology was designed to balance methodological rigour with the flexibility required for iterative exploration of emerging concepts and findings. The review followed a six-stage process: (1) planning, (2) exploratory searching, (3) successively focused searching, (4) screening process, (5) data extraction, and (6) conceptual synthesis and thematic analysis [37–39]. As assessments of study relevance may be subject to reviewer interpretation, reproducibility can be challenged by variation in how relevance is understood and applied. To enhance transparency and reproducibility, relevance criteria were specified a priori and operationalised through predefined inclusion and exclusion criteria (Table 1).

**Table 1:** Eligibility criteria for inclusion and exclusion of studies

Category	Inclusion criteria	Exclusion criteria
Study design	Empirical studies (qualitative, quantitative, or mixed-methods)	Non-empirical studies (e.g. editorials, commentaries)
Context	Healthcare organisations or healthcare service delivery settings	Non-healthcare settings
Focus	Decentralised governance, delegated authority, decision space, or managerial autonomy	Studies focusing solely on macro-level policy without relevance to service delivery
Perspective	Managerial roles, experiences, or practices (e.g. clinical directors, hybrid managers)	No managerial or organisational perspective
Organisational systems	Insights into organisational structures, support systems, or coordination mechanisms	No relevance to organisational coordination or enabling systems
Relevance to aim	Explicit or implicit contribution to understanding managers’ effective decision space	Studies not relevant to decision space or managerial autonomy
Publication type	Peer-reviewed journal articles and relevant book chapters	Grey literature (unless highly relevant) or non-scholarly publications
Language	English	Non-English publications

### Search strategy

Searches were conducted in two databases: PubMed and CINAHL (Cumulative Index to Nursing and Allied Health Literature). To ensure that the review reflected contemporary evidence, peer-reviewed studies published in the English language within the past decade (2016–2026) were included in the searches. The search strategies were developed iteratively and adapted to the indexing systems and syntax of each database. Both-free-text terms and controlled vocabulary (MeSH terms) were used when applicable corresponding to the conceptual domains of decentralisation, healthcare organisations, hospitals, management, and decision space (Supplementary file 1). The final search date for both databases was 17 March 2026 [37–39].

The search strategy was complemented by snowballing through the reference lists of relevant review articles and book chapters to identify additional studies. The conduct and reporting of the review were guided by the PRISMA-ScR (Preferred Reporting Items for Systematic Reviews and Meta-Analyses extension for Scoping Reviews) framework, together with established methodological guidance for developing search strategies and conducting scoping reviews [40–43].

### Screening process

After removal of duplicates the identified records were screened in a process based on the predefined eligibility criteria (Table 1). First, titles and abstracts were reviewed to assess relevance. Studies that did not meet the inclusion criteria or met any of the exclusion criteria were removed at this stage.

Second, full-text versions of potentially relevant studies were retrieved and assessed for eligibility. Inclusion decisions were guided by the criteria outlined in Table 1, with particular attention to relevance for decentralised governance, managerial decision-making, and organisational coordination.

**Fig. 2:**
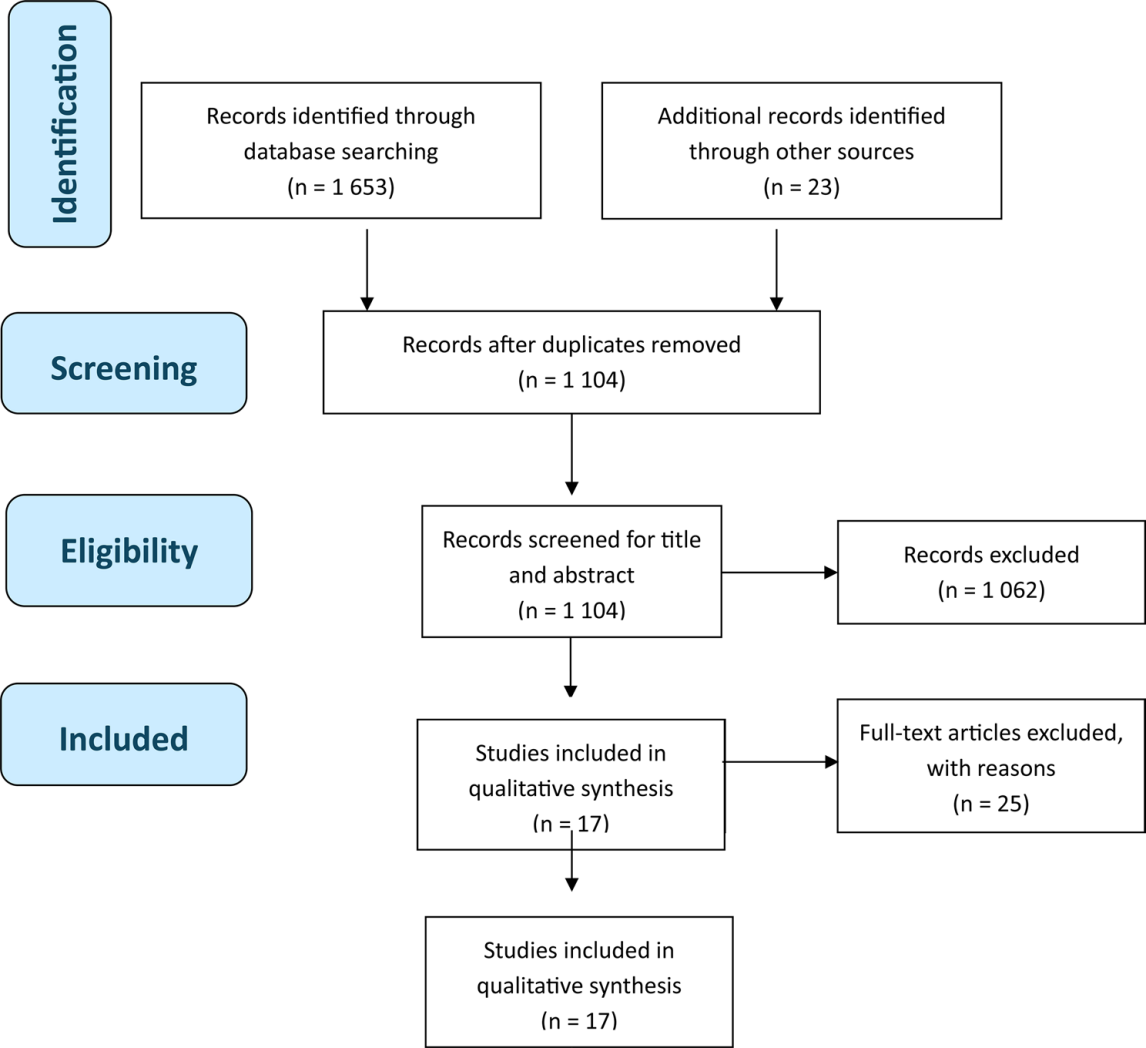
Flow diagram of the scoping review into decentralisation in healthcare delivery organisations and decision space

The screening process followed an iterative approach, allowing refinement of inclusion criteria as familiarity with the literature increased, which is consistent with established scoping review methodology. The overall selection process is illustrated in the PRISMA-ScR flow diagram (Fig. 2). Consistent with the methodological purpose of scoping reviews, no formal critical appraisal of included studies was conducted. The aim of the review was to map and conceptualize the existing literature rather than evaluate intervention effectiveness or methodological quality [43].

### Data extraction

Data were extracted from all included studies using a concept matrix developed for the review [44]. Information was extracted and organised according to study characteristics, including country and healthcare context, year of publication, study design, level of analysis, forms of decentralisation (administrative, financial, and clinical/professional), and key findings. The concept matrix served both as a structured evidence map of the included studies and as a comparative analytical tool for systematically comparing findings across studies. Through this process, recurring patterns, relationships, and variations in how decentralisation, managerial autonomy, organisational capacity, and coordination were described across different healthcare settings were identified. The concept matrix provided the empirical foundation for the subsequent conceptual synthesis and thematic analysis.

### Conceptual synthesis and thematic analysis

**Fig. 3:**
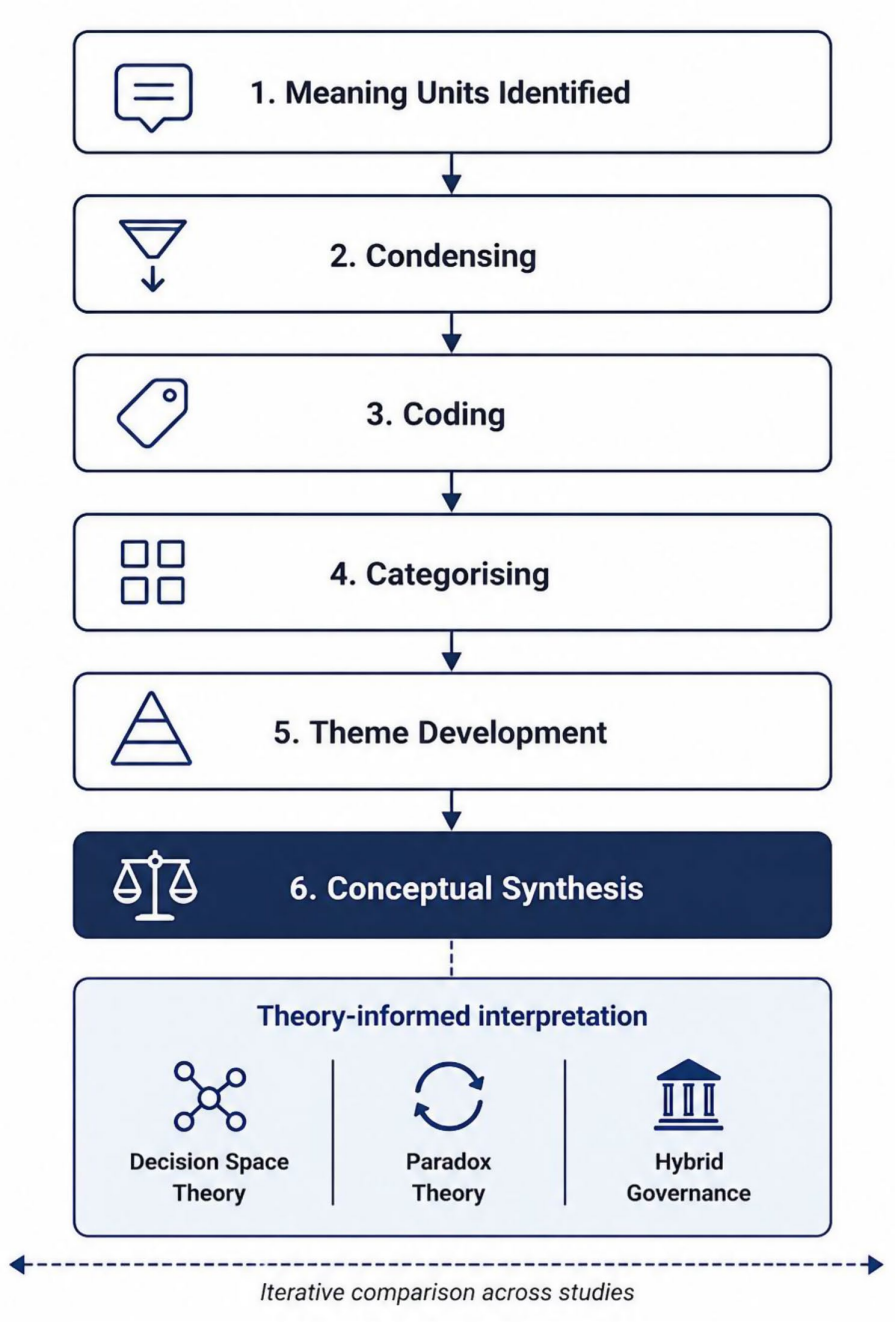
The analytical process of theory-informed conceptual synthesis. (1) Meaning units identified, (2) condensed, (3) coded, and (4) synthesised into categories before being abstracted into (5) overarching themes through iterative comparison. Themes were subsequently (6) interpreted through decision space theory, paradox theory, and hybrid governance perspectives to develop the concept of the centralisation paradox

In a second analytical step, findings relevant to the review aim were subjected to a conceptual synthesis [45]. The analysis followed an iterative process of identifying patterns across studies and developing analytical themes as shown in Fig. 3 [45–47].

Text segments describing managers’ perceptions of delegated authority, managerial autonomy, organisational capacity, accountability, governance arrangements, coordination mechanisms, relationships between central and local levels, and other findings relevant to the review objective were identified through repeated reading of the findings from the included studies and extracted as meaning units. After condensation, the meaning units were assigned descriptive codes. Similar codes were grouped into categories through iterative comparison across studies. Categories were subsequently examined for conceptual relationships and synthesized into higher-order themes [46]. This coding process was both inductive and conceptually informed. The initial coding was inductive and grounded in the empirical material. The process moved progressively from descriptive aggregation of empirical findings towards conceptual abstraction and theory-informed interpretation. During subsequent stages of analysis, emerging categories were interpreted and refined through the theoretical lenses of paradox theory [25], the decision space framework [1, 10], and hybrid management and governance perspectives [34]. The final theme structure emerged through repeated analytical and iterative refinement of conceptual boundaries between themes. The analytical themes were subsequently synthesised into four broader themes [46, 47]. These themes are presented as the main findings of the review. Through the integration and abstraction of empirical and theoretical elements, a conceptual model of the centralisation paradox within the decision space framework is developed [48, 49].

### Findings

Seventeen studies met the inclusion criteria and were included in the final analysis. The characteristics of the included studies are presented in Table 2.

**Table 2:** Concept matrix of included studies

Study	Country / context	Study type	Level of analysis	Administrative decentralisation	Financial decentralisation	Clinical / professional autonomy		Decision space / governance	Key focus
Lee T, McKee D. Int J Health Serv. 2015;45:378–397.	Costa Rica	Quantitative policy evaluation	Hospital / clinic	✓	✓	–		–	Devolution of administrative authority to hospital directors reduced costs without harming quality
Mannion R, Goddard M, Kuhn M, Bate A. Appl Health Econ Health Policy. 2005;4:47–54.	England	Empirical policy analysis	Provider organizations	✓	✓	–		✓	Incentives created by decentralising decision-making to providers
Thompson D, Snape E, Stokes C. Int J Health Plann Manage. 1999;14:19–39.	Hong Kong	Case study	Hospitals	✓	–	✓		–	HR reforms within decentralised hospital authority
London JD. Soc Sci Med. 2013;96:232–240.	Vietnam	Organisational / policy study	Hospitals	✓	✓	✓		–	Effects of hospital autonomy reforms
Aas IHM. Int J Health Plann Manage. 1997;12:103–114.	Norway	Organisational study	Hospitals	✓	–	✓		–	Coordination and innovation challenges in decentralised hospitals
Ohrling M, Solberg Carlsson K, Brommels M. BMC Health Serv Res. 2022;22.	Sweden	Qualitative case study	Healthcare provider organisation	✓	–	✓		✓	Decentralised management during COVID-19 crisis
Ohrling M. BMC Health Serv Res. 2026;26:145.	Sweden	Qualitative study	Healthcare provider organisation	✓	–	✓		✓	Hybrid management between clinical and administrative leaders
Ohrling M, Tolf S, Solberg Carlsson K, Brommels M. J Health Organ Manag. 2021;35:596–613.	Sweden	Interview study	Healthcare provider organisation	✓	–	✓		✓	Senior management perceptions of decentralisation
Ohrling M, Tolf S, Solberg-Carlsson K, Brommels M. Health Serv Manage Res. 2022;35:215–228.	Sweden	Qualitative study	Healthcare provider organisation	✓	–	✓		✓	Managerial autonomy in decentralised healthcare organization
López-Casasnovas G, McDaid D, Costa-Font J. Int Public Manag Rev. 2014.	Spain (Catalonia)	Empirical sector analysis	Hospitals / provider organisations	✓	✓	–		✓	Management autonomy of hospitals in a decentralised regional system
Chen J, et al. Health Policy Plan. 2021.	Uganda	Survey / empirical analysis	Health facilities	✓	–	–		✓	Relationship between decentralisation and facility performance
An N, et al. Risk Manag Healthc Policy. 2025.	China	Quantitative efficiency analysis	Public hospitals	✓	✓	–		–	Impact of hospital autonomy reform on efficiency
Bossert TJ, Mitchell AD. Soc Sci Med. 2011;72:39–48.	Pakistan	Empirical analysis	District health system	✓	–	–		✓	Decision space, institutional capacity and accountability
Liwanag, HJ, Wyss, K. Health Policy Plan. 2019	Philippines	Policy / governance analysis	Local health governance	✓	–	–		✓	Interaction between decision space, capacity and accountability
Seshadri SR, Parab S, Kotte S, Latha N, Subbiah K. Health Policy Plan. 2016;31:171–181.	India	Quantitative Survey study	District Health system	✓	✓	–		✓	Perceived decision space relationship between decentralisation, capacity and performance.
Goddard M, Mannion R. J Health Organ Manag. 2006;20 (1):67 − 63.	England	Empirical study	Health Provider	✓	✓	✓		✓	Coexistence of Centralisation and Decentralisation in NHS.
Witter S, van der Merwe M, Twine R, et al. PLOS One. 2024;19;e0304775.	South Africa	Qualitative multi-level case study	Provice, district, sub-district facilities	✓	–	–	✓		Decision space as interaction between authority, accountability and capacity; constraints and opportunities

Notes. Administrative decentralisation denotes delegated authority over organisational management, including staffing, work organisation, planning, and operational processes. Financial decentralisation denotes delegated authority over financial resources, including budget allocation, expenditure decisions, and financial incentives. Clinical/professional autonomy denotes discretion exercised by healthcare professionals and clinical managers regarding clinical priorities, service delivery, and professional practice. Decision space/governance denotes the range of choices available to local actors within formally delegated authority and the governance mechanisms that shape, constrain, coordinate, and hold accountable the exercise of that authority. Studies were classified according to their primary empirical focus

The included studies represented diverse healthcare contexts, organisational levels, and forms of decentralisation. Through an iterative process of conceptual synthesis, 376 meaning units were identified across across the findings sections of the included studies. These meaning units were condensed into 32 codes, grouped into 11 categories, and subsequently synthesised into four interrelated analytical themes: (1) delegated authority and experienced autonomy, (2) the role of capacity in enabling decision space, (3) persistent central coordination within decentralised systems, and (4) autonomy dependent on coordination.

The coding process and analytical development from meaning units to themes are presented in Supplementary File 2.

Table 3 summarises the analytical development from codes and categories to the four overarching themes. The table presents the categories identified across the included studies, the associated codes, the number of included studies contributing evidence to each category, and illustrative examples of key studies. By reporting the number of contributing studies, the table provides transparency regarding the evidentiary basis of the synthesis and demonstrates that the analytical themes emerged through iterative comparison of findings across multiple empirical contexts rather than from isolated observations.

**Table 3:** Categories, codes, and development of analytical themes and number of contributing studies

Theme	Category	Codes	No. of Contributing Studies	Example Studies
T1. Delegated Authority and Experienced Autonomy	Experienced autonomy	Delegated authority; Local responsiveness; Localised decision-making; Managerial discretion	11	Aas (1997); Lee & McKee (2015); Chen et al. (2021); Ohrling et al. (2021); London (2013)
	Uneven autonomy	Function-specific autonomy; De facto versus de jure autonomy	6	Bossert & Mitchell (2011); Chen et al. (2021); Witter et al. (2024)
	Outcomes of autonomy	Flexibility through autonomy; Limits of delegation	8	London (2013); Liwanag & Wyss (2019); Ohrling et al. (2022a)
T2. Organisational Capacity and Enabling Structures	Organisational capacity	Capacity enabling autonomy; Capacity development; Capacity building; Enabling infrastructure	12	Bossert & Mitchell (2011); Liwanag & Wyss (2019); Seshadri et al. (2016); Witter et al. (2024)
	Capacity–authority interaction	Authority–capacity synergy; Capacity constraints	9	Bossert & Mitchell (2011); Witter et al. (2024); Chen et al. (2021)
	Governance capacity	Accountability mechanisms; Authority–competence alignment	7	Bossert & Mitchell (2011); Liwanag & Wyss (2019); Aas (1997)
	Enabling structures	Information infrastructure; Supportive centralisation	8	Aas (1997); Ohrling et al. (2021); Thompson et al. (1999)
T3. Persistent Central Coordination within Decentralised Systems	Central coordination mechanisms	Persistent central control; Coordinating governance; Hybrid governance	10	Goddard & Mannion (2006); London (2013); Ohrling et al. (2026); Aas (1997)
	Need for integration	Coordination challenges; Goal divergence	7	Aas (1997); López-Casasnovas et al.; Goddard & Mannion (2006)
	Coordination mechanisms	Informal coordination; Coordinated decentralisation	8	Witter et al. (2024); Thompson et al. (1999); Ohrling et al. (2021b)
T4. Autonomy Dependent on Coordination	Interdependence of autonomy and coordination	Coordination dependency; Coordination paradox; Authority–capacity–accountability alignment; Dynamic decentralisation; Emergent decision space; Centralisation paradox; Autonomy–coordination balance	13	Witter et al. (2024); Bossert & Mitchell (2011); Ohrling et al. (2026); Ohrling et al. (2022b); Aas (1997); Goddard & Mannion (2006)

While Table 3 presents the analytical development of themes, Table 4 illustrates how the individual studies contributed to the four overarching analytical themes. The included studies differed in context, organisational setting, and methodological approach, yet many contributed to multiple themes. This pattern highlights the interconnected nature of autonomy, organisational capacity, coordination, and governance within decentralised healthcare systems. The table also illustrates that the conceptual development of the centralisation paradox was informed by evidence drawn from a broad range of empirical studies rather than any single source.

**Table 4:** Contribution of included studies to analytical themes

Study	T1 Autonomy	T2 Capacity	T3 Coordination	T4 Interdependence
Ohrling et al. 2026	✓	✓	✓	✓
Ohrling et al. 2022b (COVID management)	✓	✓	✓	✓
Ohrling et al. 2021 (“That’s how it should work”)	✓	✓	✓	✓
Ohrling et al. 2022a (“Managers do it their way”)	✓	✓	✓	✓
Bossert & Mitchell 2011	✓	✓		✓
Liwanag & Wyss 2019		✓		✓
Chen et al. 2021	✓	✓		✓
Seshadri et al. 2016	✓	✓		✓
Witter et al. 2024	✓	✓	✓	✓
Goddard & Mannion 2006			✓	✓
Mannion et al. 2005	✓		✓	✓
Thompson et al. 1999	✓	✓	✓	
London 2013	✓	✓	✓	✓
Lee & McKee 2015	✓			
López-Casasnovas et al.	✓		✓	✓
Aas 1997	✓	✓	✓	✓
An et al. 2025		✓		✓

The four analytical themes are presented below.

### Delegated authority and experienced autonomy

Across the included studies, decentralisation was consistently associated with the delegation of decision-making authority to lower organisational levels. Managers in decentralised healthcare systems reported increased responsibility for operational decision-making and service organisation, supporting the core assumption of decentralisation reforms that local actors are better positioned to respond to contextual needs [16, 50]. Managers reported that decentralised governance provided substantial delegated authority [14–16].

Clinical directors emphasised that proximity to clinical operations and understanding of local contexts enabled decisions better aligned with patient needs and service demands [14]. Empirical evidence further indicates that such delegated authority is often associated with improvements in responsiveness, efficiency, and service adaptation [51]. For example, decentralisation reforms in Costa Rica enabled local providers to improve efficiency without negatively affecting health outcomes [52]. Similarly, studies of decentralised healthcare management show that increased autonomy enhances organisational flexibility and responsiveness to local demands [53]. The Hong Kong study illustrated how operational authority may be decentralised while strategic interpretation remains centralised, highlighting the distinction between formal delegation and experienced autonomy [50].

However, several studies indicate that delegated authority alone does not guarantee improved performance or effective autonomy. Evidence from Uganda shows that decision space is uneven across managerial functions and does not consistently translate into improved outcomes [20]. Findings from the Swedish hybrid management study showed that delegated authority was more effectively exercised when supported by clear organisational roles, strategic alignment, and coordination structures [14]. Clinical directors reported that additional managerial levels could strengthen autonomy by facilitating coordination and providing organisational support, although poorly defined responsibilities could have the opposite effect [14].

Several studies provided concrete examples of how contextual conditions and supporting mechanisms shaped the effects of decentralisation. In the Swedish studies [14–16], managers described how delegated authority was facilitated by organisational support structures, including human resource services, financial management systems, administrative support functions, and clearly defined governance arrangements. During the COVID-19 pandemic, local managers were able to rapidly adapt services and reorganise care delivery, but their actions were supported by centrally coordinated information systems, resource allocation processes, and crisis management structures [19]. Similarly, studies from Pakistan [2], the Philippines [24], and the English NHS [54] demonstrated that the effects of decentralisation depended on institutional capacity, accountability arrangements, governance structures, and access to organisational resources. These findings suggest that delegated authority alone is insufficient to generate effective autonomy unless supported by organisational conditions that enable managers to exercise their decision space in practice.

### Organisational capacity and enabling structures

A second theme concerns the role of managerial and organisational capacity in enabling the exercise of delegated authority. Across studies, capacity emerged as a critical determinant of whether decision space could be translated into effective action. Organisational capacity was operationalised through concrete enabling structures rather than abstract organisational characteristics. While managers valued delegated authority, they consistently emphasised the importance of organisational capacity in enabling its use. For example, managers in Swedish healthcare organisations described dependence on shared electronic health record systems, centralised human resource functions, financial reporting systems, procurement processes, and administrative support services that facilitated coordination across organisational units and enabled local decision-making. These systems provided the infrastructure required to translate delegated authority into operational action and enabled managers to monitor performance, allocate resources, and coordinate care delivery effectively. Although managers had delegated authority to reorganise services and adapt care delivery to local circumstances, they remained dependent on centrally maintained information systems, administrative support functions, and organisational routines that made such decisions actionable in practice [14, 16, 19].

Seshadri et al. [55] similarly found that decentralisation did not automatically increase local autonomy. Despite formal devolution of functions under the National Rural Health Mission, district-level managers reported limited decision space in areas such as budgeting, contract management, and human resource management. The authors argued that effective decentralisation depended on sustained investments in managerial and organisational capacity to enable local actors to exercise delegated authority in practice.

Witter et al. [56] extended this argument by conceptualising decision space as an emergent property of authority, capacity, and accountability. Their findings showed that although formal authority was generally well defined, local decision-making remained constrained by shortages of staff, infrastructure limitations, fragmented information systems, and weaknesses in management support. Capacity therefore functioned not merely as a contextual factor but as a prerequisite for the effective exercise of decision space.

Empirical evidence suggests strong interdependencies between decision space and capacity. In Pakistan, higher levels of decision space were associated with stronger institutional capacity and accountability mechanisms, suggesting that these dimensions operate synergistically [2]. Similarly, research from the Philippines shows that expanding decision space without corresponding investments in capacity limits the effectiveness of decentralisation reforms [24].

Within organisations, managers highlighted that their ability to act depended on access to resources, administrative support, and infrastructure. Studies further show that system-level support, such as leadership development, organisational routines, and information systems, is essential for exercising authority effectively [14, 16]. More recent evidence indicates that decentralisation improves performance only when accompanied by sufficient capacity and governance structures [57].

The findings also highlight the complexity introduced by multiple hybrid managerial levels. While additional organisational layers may strengthen coordination and strategic alignment, they can also increase complexity and create ambiguity in roles and responsibilities when not clearly defined [14].

### Persistent central coordination in decentralised systems

Despite the emphasis on local autonomy, the analysis shows that central coordination remains a persistent and necessary feature of decentralised healthcare systems.

Several studies indicate that decentralisation reforms coexist with continued central control over key domains such as policy, financing, and administrative systems. For example, in Hong Kong, operational responsibilities were decentralised while central authorities retained control over policy interpretation and strategic direction [50]. Similarly, studies of the NHS show that decentralisation is often accompanied by mechanisms of central oversight, creating ongoing tensions between autonomy and coordination [58].

Findings from the hybrid management study further suggested that clinical directors frequently requested greater operational autonomy while simultaneously advocating stronger coordination and support from higher organisational levels. Additional managerial layers were often perceived as both a source of complexity and an important mechanism for coordination, illustrating the interdependence between autonomy and organisational integration with persistent central coordination [14].

At the organisational level, centrally coordinated systems such as digital infrastructure, financial management, and administrative processes are critical for enabling decentralised units to function effectively. For example, these included electronic health record systems, support for workforce planning and financial management, as well as governance of reimbursement mechanisms and major capital investments. Managers depend on these systems to coordinate activities, access information, and implement decisions [14–16, 19].

Evidence from crisis management further illustrates this relationship. During the COVID-19 pandemic, decentralised managers were able to rapidly adapt clinical operations, redesign patient pathways, and reallocate resources locally. However, these actions were supported by centrally coordinated structures for information sharing, resource distribution, procurement, and strategic coordination. The findings therefore suggest that local flexibility and central coordination operated simultaneously rather than as alternative governance mechanisms [19].

In the Swedish studies managers did not seek autonomy in all organisational domains. Rather, they distinguished between areas where local discretion was desirable and areas where central coordination was considered beneficial. Centralised policies regarding investments, human resources, and administrative systems were frequently perceived as necessary to support operational effectiveness and organisational coherence [16]. Senior managers described decentralisation as dependent upon a shared organisational framework that provided strategic direction, common priorities, and system-wide coordination. Local autonomy was therefore viewed as most effective when exercised within clearly defined organisational structures rather than independently of them [15].

### Autonomy dependent on coordination

The synthesis of findings reveals a consistent pattern that can be conceptualised as a centralisation paradox. Across empirical contexts, managers simultaneously value local autonomy and depend on central coordination to exercise that autonomy effectively [14, 16].

A recurring pattern across the included studies was that managers sought increased discretion over clinical and operational decisions while simultaneously expressing support for central coordination in enabling organisational domains. Centralised digital systems, human resource support, financial management structures, and administrative processes were not primarily perceived as constraints on autonomy but as prerequisites for exercising authority effectively. This pattern was observed across different healthcare systems despite considerable variation in institutional context and governance arrangements [2, 14, 16, 20, 24].

Similarly, studies of decentralisation reforms show that while local autonomy enhances responsiveness and innovation, it also introduces coordination challenges that require central governance mechanisms [59]. The coexistence of decentralisation and centralisation is therefore a persistent feature of healthcare governance [54, 58].

Findings consistently suggest that decentralisation outcomes depend on the alignment of authority, capacity, and accountability. Where these elements are aligned, decentralisation can improve performance; where they are not, it may lead to inefficiencies or unintended consequences [2, 14, 20].

Evidence from dynamic and crisis contexts further illustrates this interdependence. Decentralised governance enables flexibility and responsiveness, while central coordination remains critical for structuring information flows, resource allocation, and crisis management. Managers thus experience autonomy and coordination as complementary rather than opposing elements of governance. Local experimentation and adaptation created managerial room for manoeuvre while simultaneously providing decision-makers at higher organisational levels with more accurate and context-sensitive information for strategic planning and resource allocation [14, 19].

## Discussion

This study set out to examine how central coordination in enabling organisational systems shapes managers’ effective decision space in decentralised healthcare organisations. The findings indicate a consistent pattern in which decentralisation and central coordination appear as interdependent elements that jointly shape managerial autonomy in practice and are not best understood as opposing governance mechanisms.

Taken together, the findings challenge conventional assumptions of decentralisation as a linear redistribution of authority from central to local levels [2, 3, 60]. Instead, managerial autonomy emerges as relationally constituted, shaped through the interaction between delegated authority and enabling organisational capacity [2, 10]. This interpretation aligns with the decision space model, which conceptualises decentralisation as a multidimensional construct encompassing authority, capacity, and accountability rather than formal delegation alone [58, 59]. Consistent with prior research, delegated authority is a necessary but insufficient condition for effective decentralisation, as performance outcomes depend on adequate capacity and governance structures [61–63]. The literature further indicates that central coordination is necessary to ensure equity, efficiency, and system-wide coherence. Decentralisation may lead to variation in resource allocation and service delivery, requiring central intervention to maintain standards and reduce inequalities [64].

The findings extend this literature by demonstrating that capacity is actively produced through centrally coordinated enabling systems. Managers consistently advocate increased local autonomy in clinical and operational decision-making while emphasising the need for stable central coordination in domains such as digital infrastructure, administrative systems, and standardised processes [14, 16]. Authority thus appears to become effective only when coupled with enabling conditions that sustain managerial action.

These observations provide empirical grounding for conceptualising a centralisation paradox as a structural feature of decentralised healthcare governance. Rather than reducing the role of central authority, decentralisation appears to reconfigure it, shifting central governance from direct control towards enabling and coordinating functions. This extends previous research by showing that central coordination may expand, rather than constrain, effective decision space [3, 60, 65].

From a decision space perspective, this paradox reflects the dynamic relationship between authority and capacity: managers may possess formal authority yet remain constrained in practice without the organisational conditions required to act. Conversely, centrally coordinated systems can expand effective decision space by strengthening capacity.

Interpreted through paradox theory, decentralisation and centralisation constitute a “both–and” relationship [25, 66]. These elements appear contradictory but are suggested to be mutually constitutive and persist over time [25, 30]. Organisations must therefore continuously balance autonomy and coordination rather than resolve the tension between them [25].

The nature of central coordination is critical. Drawing on Adler and Borys’ [28] distinction, managers primarily seek enabling rather than coercive forms of centralisation, structures that support professional work and reduce uncertainty rather than constrain discretion. This distinction highlights that centralisation is not a unitary construct but varies in its effects on autonomy.

**Fig. 4:**
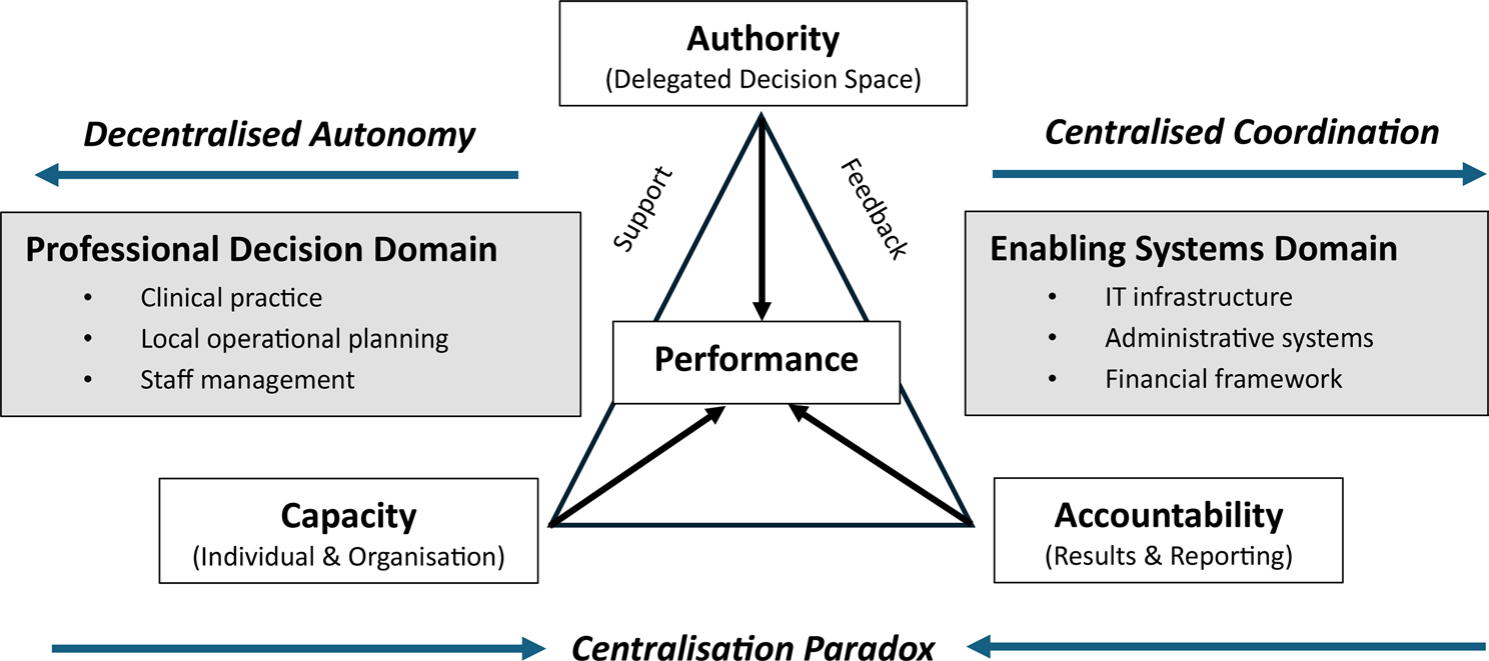
Conceptual model of the centralisation paradox within the decision space framework

Building on these insights, this study proposes a dual-domain extension of the decision space model. Based on the findings, decision space may be conceptualised as spanning two interdependent domains: (1) a professional decision domain, where authority is exercised over clinical and operational decisions, and (2) an enabling systems domain, where centrally coordinated structures provide the resources and infrastructure necessary to enact that authority. Effective autonomy emerges from the interaction between these domains rather than from authority alone (Fig. 4).

The model proposes a balance in decision-making capacity, where local autonomy as delegated authority supports the driving force for action and a feedback mechanism to the wider system. In this sense, decentralised decision space not only enables responsiveness and initiative at the operational level, but also generates continuous, context-sensitive feedback on whether centralised systems and structures are appropriately designed to support practice. Effective autonomy is therefore proposed to depend on this reciprocal dynamic, in which decision-making authority is exercised to both act and inform, ensuring that central coordination remains aligned with local realities and capable of supporting organisational performance.

### Theoretical implications

This study makes three main theoretical contributions. First, it extends the decision space model by illustrating that autonomy is co-produced by authority and enabling capacity, rather than determined by authority alone. Second, it proposes the centralisation paradox as a core mechanism in decentralised healthcare governance, linking decision space theory with paradox theory. Third, it suggests a dual-domain conceptualisation of decision space, which distinguishes between professional decision domain and enabling systems domain and highlights their interdependence. Together, these contributions shift the understanding of decentralisation from a structural arrangement to a dynamic, relational process shaped by organisational systems.

### Implications for practice

The findings of this study suggest that decentralised healthcare organisations should move beyond viewing autonomy and centralisation as opposing governance mechanisms. Instead, organisational design should explicitly recognise the interdependence between local decision space and centrally coordinated enabling systems.

For policymakers and senior management, this implies that strengthening decentralisation requires investments not only in delegated authority but also in organisational capacity, particularly in enabling domains such as digital infrastructure, administrative support, leadership training, and coordination mechanisms. Efforts to reduce central involvement indiscriminately may inadvertently weaken local autonomy if they undermine the systems required to support managerial action.

For organisational leaders, the results highlight the importance of distinguishing between coercive and enabling forms of centralisation. Central coordination should be designed to support clinical work and reduce uncertainty rather than impose rigid control. Clarifying roles and responsibilities across hybrid managerial levels is also critical to avoid ambiguity and duplication. Recognising the centralisation paradox may therefore help healthcare organisations design governance models that sustain both professional autonomy and organisational coherence.

### Limitations and future research

This study has several limitations that should be considered when interpreting the findings. First, the analysis is based on a conceptual synthesis of findings from previously published empirical studies rather than on new primary data. While this approach enables the integration of insights across multiple studies and contexts, it also means that the findings depend on the scope, quality, and methodological approaches of the included studies. Variations in research design, measurement of decentralisation, and contextual conditions across studies may limit the comparability of findings and the ability to draw generalisable conclusions.

Second, as highlighted in previous research, there is a relative lack of detailed empirical studies examining how decentralised governance operates in practice at the organisational and managerial levels. This limits the ability to fully capture the micro-level processes through which delegated authority is enacted and coordinated.

Third, the conceptualisation of the centralisation paradox developed in this study is theory-driven and interpretive. While it is grounded in empirical findings and established theoretical frameworks, it has not yet been empirically tested as a distinct construct. The extent to which the centralisation paradox can be observed, measured, and generalised across different healthcare systems and organisational contexts remains an open question.

Future research should therefore focus on empirically examining the centralisation paradox in different healthcare settings. Comparative studies across organisations and health systems could help to identify how variations in organisational design, institutional context, and governance arrangements influence the relationship between decentralisation and central coordination.

Further research is also needed to operationalise the concept of centralisation paradox and to develop measures that capture the interaction between delegated authority, organisational capacity, accountability, and central coordination. This could involve combining qualitative approaches with quantitative indicators of decision space, organisational capacity, and performance outcomes.

Finally, future studies could explore how different forms of central coordination, such as digital infrastructures, administrative systems, and performance management frameworks, affect managerial autonomy and organisational performance. Understanding which types of centrally coordinated systems enable rather than constrain decentralised decision-making would provide valuable insights for both theory development and the design of healthcare governance models.

## Conclusion

This study reconceptualises decentralised healthcare governance by proposing an extension of the decision space model through the concept of a centralisation paradox. The findings suggest that managerial autonomy does not arise solely from delegated authority but depends on the interaction between authority and centrally coordinated organisational capacity.

By conceptualising decision space as comprising both a professional decision domain and an enabling systems domain, the study illustrates how central coordination can strengthen rather than constrain local autonomy. This dual-domain perspective reframes decentralisation as a dynamic and relational governance arrangement in which autonomy and coordination coexist and mutually reinforce each other.

This reconceptualisation provides a foundation for future research on governance in complex healthcare systems and offers a framework for designing organisations that can sustain both adaptability and coordination under increasing system pressures.

## Supplementary Information

Below is the link to the electronic supplementary material.

Supplementary Material 1

Supplementary Material 2

## Data Availability

The datasets used and/or analyzed during the current study are available from the corresponding author upon reasonable request.
