# Supplementary material for: Having and eating the cake? The centralisation paradox in decentralised healthcare governance: a scoping review
Source: BMC Health Serv Res. 2026 Jul 24;26:1020. doi: 10.1186/s12913-026-15162-1 (PMC13397811; doi:10.1186/s12913-026-15162-1)
Supplement: Supplementary file 1 — Supplementary Material 1 [file 12913_2026_15162_MOESM1_ESM.docx]

**Supplementary file 1**

| **Database** | **Search strategy** | **Filters** | **Final**  **Search** | **Results** |
| --- | --- | --- | --- | --- |
| PubMed | (“Decentralization, Organizational”[Mesh] OR decentralisation[Title/Abstract] OR decentralization[Title/Abstract]) AND (“Health Services Administration”[Mesh] OR “health care organisation*”[Title/Abstract] OR hospital*[Title/Abstract] OR “health system*”[Title/Abstract]) AND (“decision space”[Title/Abstract] OR governance[Title/Abstract] OR management[Title/Abstract] OR leadership[Title/Abstract]) | Peer reviewed;  English;  2016–2026 | 17 March 2026 | n=780 |
| CINAHL | TX (decentralisation OR decentralization) AND TX (“health care organisation*” OR “healthcare organisation*” OR hospital* OR “health system*”) AND TX (“decision space” OR governance OR management OR leadership) | Peer reviewed;  English;  2016–2026 | 17 March 2026 | n = 873 |
