# Supplementary material for: Having and eating the cake? The centralisation paradox in decentralised healthcare governance: a scoping review
Source: BMC Health Serv Res. 2026 Jul 24;26:1020. doi: 10.1186/s12913-026-15162-1 (PMC13397811; doi:10.1186/s12913-026-15162-1)
Supplement: Supplementary file 2 — Supplementary Material 2 [file 12913_2026_15162_MOESM2_ESM.docx]

**Supplementary file 2**

**Coding Matrix Underpinning the Conceptual Synthesis**

| **Example**  **Condensed Meaning Unit** | **Code** | **Category** | **Theme** |
| --- | --- | --- | --- |
| Authority is transferred from central management to lower organisational levels | Delegated authority | Experienced autonomy | T1. Delegated Authority and Experienced Autonomy |
| Managers adapt services to local patient and population needs | Local responsiveness | Experienced autonomy | T1 |
| Decision-making occurs closer to clinical operations | Localised decision-making | Experienced autonomy | T1 |
| Managers report increased discretion in operational decisions | Managerial discretion | Experienced autonomy | T1 |
| Decision space varies across managerial functions | Function-specific autonomy | Uneven autonomy | T1 |
| Formal authority differs from authority exercised in practice | De facto versus de jure autonomy | Uneven autonomy | T1 |
| Autonomy improves flexibility and innovation | Flexibility through autonomy | Outcomes of autonomy | T1 |
| Delegation alone does not guarantee improved performance | Limits of delegation | Outcomes of autonomy | T1 |
| Organisational capacity determines whether authority can be exercised effectively | Capacity enabling autonomy | Organisational capacity | T2. Organisational Capacity and Enabling Structures |
| Local actors require resources, competence and support systems | Enabling infrastructure | Organisational capacity | T2 |
| Responsibility contributes to managerial learning and competence development | Capacity development | Organisational capacity | T2 |
| Capacity-building is required for decentralisation reforms to function effectively | Capacity building | Organisational capacity | T2 |
| Capacity and authority reinforce one another | Authority–capacity synergy | Capacity–authority interaction | T2 |
| Weak capacity constrains the use of delegated authority | Capacity constraints | Capacity–authority interaction | T2 |
| Accountability mechanisms support effective use of authority | Accountability mechanisms | Governance capacity | T2 |
| Authority should be aligned with competence and capability | Authority–competence alignment | Governance capacity | T2 |
| Information systems support local decision-making | Information infrastructure | Enabling structures | T2 |
| Central support functions facilitate local implementation | Supportive centralisation | Enabling structures | T2 |
| Strategic control remains centralised despite decentralisation reforms | Persistent central control | Central coordination mechanisms | T3. Persistent Central Coordination within Decentralised Systems |
| Central governance maintains system-wide coherence and equity | Coordinating governance | Central coordination mechanisms | T3 |
| Decentralisation introduces risks of fragmentation | Coordination challenges | Need for integration | T3 |
| Local autonomy coexists with central oversight | Hybrid governance | Central coordination mechanisms | T3 |
| Organisational units may pursue divergent goals | Goal divergence | Need for integration | T3 |
| Informal relationships facilitate collaboration and coordination | Informal coordination | Coordination mechanisms | T3 |
| Reporting systems, communication and common goals remain necessary | Coordinated decentralisation | Coordination mechanisms | T3 |
| Managers value autonomy but depend on central support systems | Coordination dependency | Interdependence of autonomy and coordination | T4. Autonomy Dependent on Coordination |
| Central systems both enable and constrain autonomy | Coordination paradox | Interdependence of autonomy and coordination | T4 |
| Authority, capacity and accountability must align | Authority–capacity–accountability alignment | Interdependence of autonomy and coordination | T4 |
| Flexibility during crises depends on coordinated support systems | Dynamic decentralisation | Interdependence of autonomy and coordination | T4 |
| Decision space emerges through interactions among governance dimensions | Emergent decision space | Interdependence of autonomy and coordination | T4 |
| Effective decentralisation requires balancing autonomy and control | Centralisation paradox | Interdependence of autonomy and coordination | T4 |
| Autonomy and coordination operate simultaneously rather than as opposites | Autonomy–coordination balance | Interdependence of autonomy and coordination | T4 |
